# Depression in Sardinian immigrants in Argentina and residents in Sardinia at the time of the Argentinian default (2001) and the Great Recession in Italy (2015)

**DOI:** 10.1186/s12888-017-1226-1

**Published:** 2017-02-08

**Authors:** Mauro Giovanni Carta, Michela Atzeni, Silvia D’Oca, Alessandra Perra, Ernesto D’Aloja, Maria Veronica Brasesco, Maria Francesca Moro, Luigi Minerba, Federica Sancassiani, Daniela Moro, Gustavo Mausel, Dinesh Bhugra

**Affiliations:** 1 Department of Public Health, Clinical and Molecular Medicine, University of Cagliari, Cagliari, Italy; 2 Universidad del Museo Social Argentino, Buenos Aires, Argentina; 3 King’s College, London, UK

## Abstract

**Background:**

The aim of this study is to measure in two samples of Sardinian immigrants in Buenos Aires and representatives of the population in Sardinia the prevalence of depressive symptoms at the time of an economic crisis in Sardinia and to compare these results with those collected at the time of a similar crisis in Argentina more than 10 years before.

**Methods:**

Observational study. The associations of Sardinian immigrants in Buenos Aires provided the lists of families of Sardinian origin. A random sample of one fifth of registered families was selected. The sample of a study carried out in Sardinia was used as the control. The results were compared with those of the previous study performed in 2001–2002. The Patient Health Questionnaire (PHQ9) was used for the screening of depression.

**Results:**

The Sardinian immigrants show a lower rate of scoring positively on PHQ9 (i.e. less risk of being depressed) and reach statistical significance after standardization (8.7% vs. 13.1%, *P* = 0.046). Young women (≤40) are at higher risk. On the contrary, the risk of depression was higher in Sardinian immigrants in Argentina during the 2001–2002 crises.

**Conclusion:**

The study indicates a risk for depressive episodes linked to the fallout of the economic crisis (in Argentina in 2001–2002, in Sardinia in 2015) and specifically more in females than in males. Due to the associated socio-demographic risk factors, these results could be interpreted as due to an increase in non-bipolar depression.

## Background

Migration exists from the origin of humankind: the causes of migration were obviously about survival but it is also likely that a search for novelty or reasons having to do with politics and religion may well have contributed [1].

Several studies about the risk of psychopathology in migrants have confirmed Murphy’s hypothesis [2] that migrants cannot be considered a homogeneous group with respect to psychopathological risk. Psychosocial studies have thus tried to identify the factors which may determine an increased risk of psychopathology in specific migrant groups in specific host conditions [3]. In this framework, the following variables have been studied: motivation to migrate (which Phillip Rack had subdivided in settlers, refugees and “gastarbeiters,” [4]); distance from the culture of the host country (religion, language, and so on [5]); resilience and attitude to develop mediating structures and an evolving identity [6–8]; factors related to legal residential status [1]; acceptance-reaction phenomena in the host country [9].

Our previous findings from Sardinia and Argentina revealed a high risk of Major Depressive Disorders in Sardinian immigrants in Argentina compared to Sardinian residents in Sardinia [10] Women older than 45 years who had emigrated were at a higher risk compared with Sardinian women of the same age living in Sardinia [10].

The findings of this survey were quite different from those of another survey conducted on Sardinian immigrants in Paris in the 1990s [11]. The Sardinian immigrants showed a higher risk of depression compared to Sardinian residents in Sardinia but, contrary to Argentina, a strong association was found in young males rather than females [11]. The timings of these surveys were related to economic factors. In Argentina it was the period of economic slow-down whereas in France it was a time of economic growth that may explain variation in rates between the two genders.

These differences were interpreted by recalling the hypothesis of “goal striving stress” [12]: migration selects people (especially men) who are hyperactive and seek novelties, but they may be vulnerable to stress due to the effort to achieve determined social goals if they lack the means for achieving such goals [13]. This appears to be typical of modern western societies in periods in which the economy is expanding; in this framework the possibility of improving one’s economic status may be counterbalanced by the stress of achieving objectives which the individuals may set for themselves. But they are also very strongly influenced by external factors such as the media. However, the reality is often different since these goals are difficult to reach by people in the most disadvantaged circumstances.

The findings of the study in Paris [11] assumed that the excess of depression in young people was the result of an excess of bipolar disorders also because of association with anxiety disorders, substance abuse and recurrent brief depressive conditions [14].

Similar factors may well explain the excess of suicides in young males during the Irish economic boom (1980–2000) [15]. At that time, Ireland was the European country with the highest increase in suicides in males and the only European country with a higher suicide rate in young males than in elderly men [16].

Historically, the peak of Sardinian emigration to Argentina was in the late 1950s and early 1960s. It is possible that women were more likely to be secondary migrants and that may have made them more vulnerable to depression when exposed to conditions of economic downturn [13], according to John Bowlby’s [17] model of compulsive hyper-responsibility.

Bipolar depression affects males and females equally, although Major Depressive Disorder is 3 times more common in females. This risk of Major Depression may also be related to crises. In a study we found in the same sample of this study that rates of bipolar disorders were higher in migrants than in residents in Sardinia [18].

However, studies comparing the same populations over time found that the impact of the economic crisis on the mental health of the population was pervasive. A community survey repeated in Greece with the same methodology over time found at the peak of an economic crisis in 2011, a one-month prevalence rate of major depression of 8.2%, as compared to 3.3% before the crisis in 2008 [19].

Thus we decided on a new approach, using a quasi-experimental design, comparing two different populations (Sardinian migrants in Argentina and Sardinians resident in Sardinia) exposed to economic crises at different points in time.

Some simple data may describe the extent of the two periods of crisis in which the two studies were conducted.

In 2001–2002 (during the first survey) the Argentinian economy suffered the sharpest decline since 70 years before; Argentina had defaulted on its debt and the Argentinian currency value had depreciated by 70%. The per capita gross domestic product (GDP) declined from 7.17 thousand US dollars in 2001 to 4.70 thousand in 2004, with a dramatic collapse in 2001–2002 just at the time of the survey [20].

During this first survey the mean Italian per capita GDP increased from 20.40 thousand US dollars in 2001 to 31.20 in 2004. The ratio GDP / cost of living was better than in the UK and France and similar to Germany [20].

In 2015, during the second survey, Argentina, as in the rest of the world, suffered from the so-called “The Great Recession”, which exploded in 2008 and which had its peak in the US in 2009, thus Argentina’s economy was not stable, but the GDP reached 12.80 thousand US dollars, more than double compared to the crisis period of less than 15 years before. Despite the crisis Argentinians were still benefiting from a relative wellbeing compared to what they had experienced from fifteen to ten years earlier.

At the time of the second survey, Italy was one of the European states that was continuing to suffer most from the consequences of the “Great Recession”, and was experiencing the most severe economic crisis since the end of World War II: the GDP per capita reached 40.60 in 2008 but dramatically decreased to 29.80 in 2015 [20]. In the same period, in Italy many social and economic indicators revealed the weighty consequences of the crisis, for example the unemployment rate in 2015 was 11.6%, with the highest peaks in the south and islands (specifically in Sardinia it was 17%) [21].

This study will measure the prevalence of depressive symptoms in a sample of Sardinian emigrants in Argentina and in the community in Sardinia and compare these results in the current economic condition (characterized by a serious economic crisis in Italy) with previous economic downturns in Argentina. Our hypothesis is that while in the past rates had been higher among Sardinian immigrants to Argentina, now one expects them to be higher among residents in Sardinia. We also hypothesized that rates of depression may be higher in Sardinian women.

## Methods

### Design

Observational transversal study (“community survey”) conducted on a sample of the general population of Sardinian residents in Buenos Aires. These findings will be compared with prevalence in the Sardinian population using the same method and screening tools [22]. The results of the present study will be compared with those of the previous cited study [10].

### Sample

The research was conducted in the city of Buenos Aires and involved adults over the age of 18.

The associations of Sardinian emigrants in Buenos Aires provided the lists of individuals who had migrated from Sardinia. A random sample of a total of one fifth of registered families was selected.

Each household was contacted by phone to verify in advance if the household included Sardinian immigrants of first- or second-generation (if they were children of both parents from Sardinia); only such people were invited to participate in the research.

After verification, preliminary consent to participate was obtained if they agreed to participate. Brief preliminary information about name, age, the length of the migratory path and the place of origin was collected and then a face-to-face meeting was arranged. Depending upon the preference and convenience of the individuals, interviews were conducted at home, at the University (“Universidad del Museo Social Argentino”) or at the Association of Sardinians (“Sardi Uniti d’America”) offices.

### Sample of the Survey in Sardinia

From November 2011 to August 2012, a community survey was conducted on a random sample of the Sardinian population, involving people aged 16 years and older, living in private households (from this survey only people aged 18 years and older were analyzed). For sampling, the Sardinian population was broken down into 16 strata on a geographic basis representing the capital cities of the eight provinces plus villages with fewer than 5,000 inhabitants within each province. The rate of adherence to the study was 51% of those who were eligible for interviewing. In the case of the previously published research [22] data from a sub sample chosen thanks to a post-strata saturation method that subdivided it into 10 cells according to sex and five age groups for each province were used. This was done to ensure that the number of each sub-sample from a province corresponded to the proportion of the Sardinian population living in that province (Italian National Institute of Statistics (ISTAT), 2011). In this paper, in which it is planned to standardize the sample of the Sardinian population with the comparison sample (Sardinian immigrants), we chose to use the total of the interviewed aged 18 years and older. Thus the sample of Sardinians living in Sardinia is composed of 1502 subjects (728 male 48.4%, Table 1).

**Table 1:** Study Samples

	Sardinians in Argentina (306)	Sardinians in Sardinia (1502)
% Male	47.7	48.4 (✗2 = 0.87, 1df, *P* = 0.35)
% ≤40 years old	14.4	34.2 (✗2 = 46.90, 1df, *P* < 0.0001)
% Degree level	38.6	23.4 (✗2 = 30.54, 1df, *P* < 0.0001)
First Generation	48 (15.7%)	

### Instruments

The demographic information collected included gender, age, residence, education, marital status, presence of children, work and migration history. It was similar to that used in the previous survey conducted in 2001 [10].

The Patient Health Questionnaire (PHQ9) [23] was used to assess depression using the Italian version [24] and the Spanish version [25]. The PHQ9 is a self-administered or phone-administered questionnaire well established as a tool for screening depression or for the diagnosis of depressive symptoms. It is relatively easy to use and gives scores to each of the nine DSM-IV-TR criteria for major depressive disorder, proposed in the form of questions; as a severity measure, the scores of each item can range from “0” for complete absence to “3” for almost every day, with “1” for a few days and “2“for more than half of days. The value resulting from the sum of the scores assigns ranks within the following ranges for interpretation: from 0 to 4 for a minimal depression, 5 to 8 mild, 9 to 14, moderate 15–19 moderately severe and from 20 to 27 severe. Major Depressive Disorder (MDD) is diagnosed if five or more of the nine criteria-symptoms were present for at least more than half the days (score equal to or greater than 2) in the preceding two weeks and one of the symptoms is depressed mood or anhedonia. Both the Italian and Spanish versions of PHQ9 have been evaluated for use in the general population [25, 26].

The ANTAS- SCID interview [27] as well as the simplified CIDI interview – Italian Version [28] were administered to two groups of 10 individuals positive and 40 negatives to PHQ 9 in the immigrants sample to ascertain the validity and the gold standard for assessment.

### Statistical Analysis

The statistical analysis involved the following:

1. The data of the present study were compared with the “Loss of Sadness in Sardinia” study [22]. Those above and below 40 were grouped and compared along with gender. The total sample of emigrants in Argentina was compared with the total Sardinian sample, (both using unweighted samples and after indirect standardization by age and sex in accordance with the four previously cited cells).
2. Risk of depression was also compared between the Sardinian immigrants in Argentina and the Sardinian residents in Sardinia, considering among emigrants the variable of being of first generation (born in Sardinia) or second generation (born in Argentina). This analysis was conducted by comparing the data with the whole sample of residents, with only residents over 40 (the first-generation immigrants were all over the age of 40) and by gender.
3. We then compared our results on the current rates of depression between immigrants and residents in Sardinia (after direct standardization).
  Statistical significance was calculated on Tables 2 × 2 with the *χ*2 test. The measures are expressed as odds ratio, confidence intervals at 95% (OR, 95% CI) and were calculated using Miettinen’s method [29].

## Results

Three hundred and sixty-seven individuals were originally selected from the lists of Sardinian clubs in Buenos Aires and approached, but 61 (16.6%) either were not available, could not be traced or refused to participate. The final sample of this survey thus consists of 306 individuals of Sardinian origin (either first or second generation). Demographically and gender-wise, those who participated and those who did not were similar.

Table 1 compares Sardinian immigrants to Argentina with the sample of the study “Loss of Sadness in Sardinia” [26] on gender, age and literacy.

Sardinian immigrants were older and were more likely to be graduates with a statistically significant difference.

Table 2 analyzes the frequency of being screened as positive for depression at PHQ9 comparing Sardinian immigrants and the Sardinian residents in Sardinia (using the data bank of the study “The Loss of Sadness in Sardinia”). The sample of the Sardinians who migrated to Argentina shows a lower rate of positivity to PHQ9 (i.e. less risk of being depressed) and reaches statistical significance after standardization weighting for age and sex (8.7% vs. 13.1%, Chi square = 4.0, 1df, P = 0.046, OR = 0.66, 95% CI 0.41–0.99). Young migrant women were more likely to score positive on PHQ9 in comparison to those resident in Sardinia, with statistically significant differences.

**Table 2:** Point Prevalence of positivity at screening for depression by PHQ9 in the Sardinian immigrant sample and the Sardinian residents in Sardinia (database “Loss of Sadness” survey)

Age	N(%) Sardinians in Argentina	N(%) Sardinians in Sardinia	Statistical Comparisons
≤40 Male	1 (8.3)	21 (8.2)	Chi square with Yates correction = 0.001, 1df, *P* = 0.999, OR = 0.093, 95%IC 0.04–7.53
≤40 Female	0 (0)	48 (18.6)	Chi square = 9.32, 1df, *P* < 0.002, OR = 0.00, 95%IC 0.00–0.52
>40 Male	10 (7.5)	38 (8.1)	Chi square = 0.260, 1df, *P* = 0.610, OR = 0.82, 95%IC 0.36–1.83
>40 Female	23 (17.8)	90 (17.4)	Chi square = 0.05, *P* = 0.823; OR = 0.94 CI95% 0.55–1.61
Male	11 (7.5)	59 (8.1)	Chi square = 0.06, 1df, *P* = 0.81 OR = 0.92; IC95% 0.44–1.92
Female	23 (14.2)	138 (17.8)	Chi square = 0.16, 1df, *P* = 0.28 OR = 0.76; IC95% 0.46–1.27
≤40 (M + F)	1 (2.2)	69 (13.4)	Chi square = 3.88, 1df, *P* = 0.04; OR = 0.17; IC95% 0.01–0.99
>40 (M + F)	33 (12.5)	128 (12.9)	Chi square = 0.03, 1df, *P* = 0.86 OR = 0.94; IC95% 0.62–1.47
Total	34 (11.1)	197 (13.1)	Chi square =1.13, 1df, *P* = 0.31 OR = 0.82, 95%IC 0.54–1.22
Total after direct standardisation	27.9 (8.7)	197 (13.1)	Chi square =4.0, 1df, *P* = 0.046 OR = 0.66, 95%IC 0.41–0.99

Table 3 compares the current results with those of the previous study in 2001–2002 concerning the point prevalence of depressive episodes (CIDI-S - DSM-IV) between Sardinian immigrants in Argentina and Sardinians resident in Sardinia [after direct standardization]. The time trend showed a reversal of the risk of depression which was greater in Sardinian immigrants in Argentina during the 2001–2002 crisis, while during the current economic crisis in Italy, the risk was found to be higher in the Sardinians resident in Sardinia.

**Table 3:** Comparison of the findings of the current research with those of the study carried out in 2001–2002 [after direct standardization]

	Sardinians in Sardinia	Sardinians in Argentina	Chi square 1df;	*P*	OR Immigrants
2001-2002 (CIDI)	6.1%	12.4%;	10.56	*P* < 0.001	2.19 CI95% 132–3.64
2015 (PHQ9)	13.1%	8.7%	4.0,	*P* < 0.05	OR = 0.66, 95%IC 0.41–0.99

Table 4 illustrates the frequencies of depression among the Sardinian immigrants and the Sardinian residents, using generations as variables. Although first-generation immigrants show a reverse trend to greater association with depression than Sardinians of Sardinia, the differences are not statistically significant.

**Table 4:** First vs Second Generation migrants

	Sardinians in Argentina I generation	Sardinians in Argentina II generation	Sardinians in Sardinia	Sardinians in Sardinia vs First	Sardinians in Sardinia vs Second
Male	2 (9.5)	9 (7.2)	59 (8.1)	Chi square with Yates = 0.02, 1df, *P* = 0.98	Chi square with Yates = 0.03, 1df, *P* = 0.86
Female	5 (18.5)	18 (13.4)	138 (17.8)	Chi square with Yates = 0.01, 1df, *P* = 0.99	Chi square = 0.52, 1df, *P* = 0.21;
>40 (M + F)	7 (14.6)	26 (12.1)	128 (12.9)	Chi square with Yates = 0.11, 1df, *P* = 0.35	Chi square with Yates = 0.10, 1df, *P* = 0.75
Total	7 (14.6)	27 (10.4)	197 (13.1)	Chi square with Yates = 0.09, 1df, *P* = 0.77;	Chi square with Yates = 1.44, 1df, *P* = 0.23

Table 5 shows the data related to the internal quality control study on the accuracy of the screener compared to clinical diagnosis as Gold Standard.

**Table 5:** Result of the quality control study on the accuracy of the PHQ9 Cut-off 7; Gold Standard ANTAS-SCID and CIDIS diagnosis of DSM-IV Current Major Depressive Disorder

	ANTAS-SCID	CIDI-S
Sensitivity	72%	70%
Specificity	95%	95%
Predictive Positive Value	80%	87%
Predictive Negative Value	95%	93%

## Discussion

Our study found a higher frequency of depression in Sardinians residing in Sardinia than in Sardinians resident in Argentina, the risk being higher in young women. Unlike the research conducted at the height of the economic crisis in Argentina in 2001–2002, which showed a higher frequency of depression in Sardinians in Argentina compared to Sardinians resident in Sardinia, the present results indicate that in the midst of a serious economic crisis in Sardinia the Sardinian residents in Sardinia were found to be at higher risk of depression. Similar to Argentina in 2001–2002, women showed the highest risk but in Sardinia in 2015 the rates were higher in younger women.

It would appear that such an environment-dependent risk in the two conditions (Argentina in 2001–2002, Sardinia in 2015) appears more and particularly specific to women than to men.

With a quasi-experimental design, as demonstrated by repeated studies over time in the same populations in Greece [30] and confirmed by systematic reviews conducted on population studies of several countries [31], these results confirm that crises have a devastating impact on the psychological health of populations and cause an increase in rates of major depressive disorder.

These data also confirm to some extent the aforementioned hypothesis of Murphy [2]; according to this researcher migration does not represent a risk factor “in itself” but only under certain specific stressful conditions of context. In fact, to a certain degree the data show that the current living situation has a strong impact that might be even stronger than a person’s migratory status or cultural background.

In accordance with the Greek studies [30], our study shows that in the two different crisis situations (Argentina 2001–2002; Sardinia 2015) women surprisingly show a higher increase in risk.

However, the fact that risks linked to crises differ with gender was also highlighted by a study that showed an increase in suicides associated with crises, whose co-factor was the increase in alcohol abuse only in men, even though both alcohol abuse and suicide were found to have increased also in women [32].

The association with different factors related to gender in times of crisis was also shown in a study that found a major risk in males with loss of employment than in females with loss of employment, but at the same time the study also showed that employed females aged 50 and over with a low skilled work level were at higher risk than employed males [33].

The same studies in Greece, while showing a high general increase in depression rate in women [30], related to the onset of the crisis, showed that young employed males were more at risk than young employed women [34].

These findings raise the question of whether the different risk factors related to gender may be due to the fact that males and females are exposed differently to various kinds of mood disorders.

From this point of view, this study may provide additional information owing to the quasi-experimental model.

As is known, those who suffer from bipolar disorder are affected for a much longer portion of their lives in the phases of depression than in manic ones [35]. It is therefore likely that many studies on the epidemiology of depressive disorders may be diagnosed as Major Depressive Disorder in some cases of Bipolar Depression because they are performed by instruments such as structured clinical interviews unable to differentiate bipolar and unipolar depressive episodes [36, 37].

These considerations have relevance in the light of the results of another study by our group that shows higher frequencies of bipolar disorders in the same sample of Sardinian immigrants in Argentina [18]. In fact, in a population with a higher frequency of individuals with lifetime manic episodes we found a lower point prevalence rate of depression (currently but not in 2001) compared to Sardinians resident in Sardinia who show a lower lifetime prevalence of manic episodes. However, it is known that bipolar depression shows a female/male ratio close to 1 to 1, while in major depressive disorder the ratio is 3 to 1. Thus, in both circumstances (Sardinia in 2015 and Argentina in 2001–2002) the difference between the rates of depression may be the result of a higher incidence of non-bipolar depression (which particularly affects women) in both crises.

The results of our study appear consistent with the hypothesis that the risk of hyperthymia/bipolarity is less dependent on environmental factors than on non-bipolar depression or otherwise dependent on different determinants, where the crises in bipolar disorders are related more to factors such as the aforementioned “goal striving stress” and non-bipolar depression more related to losses [38]. It must however be clear that our hypothesis is a heuristic one and its purpose is only to stimulate attention and new research.

Our study cannot confirm the hypothesis that was advanced on the basis of the results of the first study in 2001–2002 concerning a possible selection of first-generation migrant women with characteristics of hyper-compulsive liability according to John Bowlby’s theory [17] and thus with a higher risk of depressive disorders. The first generation of women did not show a raised risk compared to residents in Sardinia which diverged from the second-generation migrant women. Although the analysis was necessarily conducted on a small sample (27 women) in which we found a “reverse” trend of risk (higher than in the residents in Sardinia) but the difference did not reach statistical significance.

Varying vulnerability related to age in women during the crisis in Sardinia (the younger ones were at risk) and in those during the crisis in Argentina (in which adult women were at risk) may be due to lifestyle changes in a changing society and may have lowered the age at onset of depressive disorders. One possible (complementary) explanation may be that the crisis in Sardinia mainly affects young people who are likely to be unemployed.

### Limitations

This study has various limitations. Firstly, duration of immigration will affect acculturation and therefore relevant responses on screening.

The Sardinians identified by the clubs cannot be truly representative of all Sardinian migrants as the Argentine registry office changed the names of immigrants of Sardinian origin running away from major conflicts and sometimes from ties they wished to forget at the time of their arrival at the end of the Second World War. Thus there may be a selection bias. The sense of shame may have prompted the Sardinians who felt they had received inadequate social success to isolate themselves from the official communities of the Sardinians and thus cannot be identified.

A limitation is due to the fact that the data have been standardized for gender and age, but not for educational attainment, in which differences between the two samples were also observed.

Another limitation is the insufficient reference information about normative data in the Argentine population of the scores of the tools used in the survey.

The 2001–2002 study used a different diagnostic tool (CIDIS-Simplified), but our quality control has shown that the screening tool adopted in our search has good accuracy when compared to the same interview used in 2001–2002 as the gold standard. Although they do not overlap perfectly, the results are at least comparable, because the studies in Sardinia and Argentina, conducted in two different periods, adopted the same instruments at the same times (CIDI-S in 2001–2002 and PHQ9 in 2015).

## Conclusions

The study indicates a risk of Depressive Episodes linked to the fallout of the economic crisis (in Argentina in 2001–2002, in Sardinia in 2015) and specifically more in females than in males. Due to the associated socio-demographic risk factors, these results could be interpreted as due to an increase in non-bipolar depression. Further studies on larger samples of migrants and other crisis scenarios will test the hypotheses generated by this study.
